# LncRNA LOC100506178 promotes osteogenic differentiation via regulating miR-214-5p-BMP2 axis in human bone marrow mesenchymal stem cells

**DOI:** 10.7717/peerj.8909

**Published:** 2020-04-15

**Authors:** Lina Li, Jie Fang, Yi Liu, Li Xiao

**Affiliations:** 1 Geriatric & VIP Department, Sichuan Academy of Medical Sciences & Sichuan Provincial People’s Hospital, Chengdu, China; 2 State Key Laboratory of Oral Diseases, National Clinical Research Center for Oral Diseases, West China Hospital of Stomatology, Sichuan University, Chengdu, China; 3 Department of Stomatology, Sichuan Academy of Medical Sciences & Sichuan Provincial People’s Hospital, Chengdu, China

**Keywords:** LOC100506178, hBMSCs, osteogenic differentiation, miR-214-5p, BMP2

## Abstract

Osteogenic differentiation is an important role in dental implantation. Long no coding RNAs (lncRNAs) are a novel class of noncoding RNAs that have significant effects in a variety of diseases. However, the function and mechanisms of LOC100506178 in osteogenic differentiation and migration of bone morphogenetic protein 2 (BMP2)-induced osteogenic differentiation of human bone marrow mesenchymalstem cells (hBMSCs) remain largely unclear. BMP2 was used to induce osteogenic differentiation of hBMSCs. Quantitative real time PCR (qRT-PCR) was used to examine the expression of LOC100506178, miR-214-5p, Runt-related transcription factor 2 (RUNX2), Osterix (Osx), and Alkaline Phosphatase (ALP) in BMP2-induced osteogenic differentiation of hBMSCs. The function of LOC100506178 and miR-214-5p was explored in vitro using Alizarin Red S Staining, ALP activity, as well as in vivo ectopic bone formation. Luciferase reporter assay was performed to assess the association between LOC100506178 and miR-214-5p, as well as miR-214-5p and BMP2. The miR-214-5p sponging potential of LOC100506178 was evaluated by RNA immunoprecipitation. In the present study, the expression of LOC100506178 was found to be increased in BMP2-induced osteogenic differentiation of hBMSCs, accompanied with decreased miR-214-5p expression and increased RUNX2, Osx and ALP expression. LOC100506178 significantly induced, while miR-214-5p suppressed the BMP2-induced osteogenic differentiation of hBMSCs. Mechanistically, LOC100506178 was directly bound to miR-214-5p and miR-214-5p targeted the 3′-untranslated region of BMP2 to negatively regulate its expression. In conclusion, our data indicate a novel molecular pathway LOC100506178/miR-214-5p/BMP2 in relation to hBMSCs differentiation into osteoblasts, which may facilitate bone anabolism.

## Introduction

Dental implantation has become the optimal choice for edentulous patients due to its advantages of comfortability, fine appearance and high success rate (Van Velzen et al., 2015). However, with the existence of maxillary sinus and decreasing height of residual alveolar bon, clinicians are frequently confronted with the insufficiency of vertical bone height in the posterior maxilla (Javed & Romanos, 2015). In particular, the poor bone quality of some patients who have lost their teeth for a long time makes it more difficult to perform implant restoration in this region. Therefore, it is difficult to obtain satisfactory initial stability of implants in the maxillary posterior region, and the failure rate is higher than that any other regions. Nowadays, with the improvement of maxillary sinus floor elevation (MSFE), this technology effectively addresses the problem of insufficient bone volume, and greatly widens the application of implant restoration (Al-Almaie, Kavarodi & Al Faidhi, 2013).

Human bone marrow mesenchymalstem cells (hBMSCs), a group of multi-potent cells, possess stem cell properties and can differentiate into various cells, including osteoblasts, adipocytes and chondrocytes et al. (Chamberlain et al., 2007; Mao & Prockop, 2012). A variety of cytokines, growth factors and signal pathway are reported to involved into the osteogenic differentiation process of in hBMSCs (James, 2013). Bone morphogenic proteins (BMPs) signaling has been found to be the most potential osteoinductive factor in the osteogenesis of hBMSCs (Tang et al., 2011). In recent decades, it has been demonstrated that long non-coding RNAs (lncRNAs) are significantly altered during the osteogenic differentiation (Song et al., 2015). For instance, Wang et al. (2017) revealed that lncRNA MEG3 regulated the expression of miR-133a-3p, and suppressed osteogenic differentiation of BMSCs and contributed to postmenopausal osteoporosis (PMOP). Peng et al. (2018) showed that anti-differentiation ANCR plays as a competitive endogenous RNA (ceRNA) functions as a suppressor of osteogenic differentiation through via sponging miRNA-758. Zhang et al. (2019) found that lncRNA MSC-AS1 positively regulated osteogenic differentiation of BMSCs by acting as a ceRNA to regulate BMP2 expression through sponging miR-140-5p. These positive and negative regulations of lncRNAs indicate a great role in controlling the differentiation state of stem cells.

Previous microarray research identified a novel lncRNA LOC100506178 displayed a significant increase in the BMP2-induced hBMSCs (Zhang et al., 2017). Moreover, bioinformatics prediction shows miR-214-5p was the potential target of LOC100506178. Nevertheless, the function of LOC100506178 and miR-214-5p and the underlying regulatory mechanisms between them are still elusive in osteogenic differentiation of hBMSCs. In this study, we triggered the osteogenic differentiation process in hBMSCs by incubation with BMP2 in vitro and induced ectopic bone formation in vivo to investigate the function role of LOC100506178 in osteogenic differentiation of hBMSCs. We further explored the regulatory mechanisms between LINC00968 and miR-3658 during osteogenic differentiation of hBMSCs. These findings will provide a potential molecular therapeutic strategy for dental implant restoration.

## Material and Methods

### Cell culture and osteogenic differentiation

Human bone marrow mesenchymal stem cells (hBMSCs) were obtained from Sciencell (Carlsbad, CA, USA) and cultured in Mesenchymal Stem Cell Medium (MSCM, Sciencell, CA, USA) supplemented with 10% fetal bovine serum (FBS) and 1% penicillin/streptomycin solution at 37 °C under humid conditions with 5% CO_2_. For osteogenesis differentiation, hBMSCs were seeded into six-well plate at a density of 2 × 10^4^ cells per well under normal culture condition. After 2 days, the cells were cultured with medium with 100 ng/mL of BMP2 (Invitrogen, Carlsbad, CA, USA) for osteogenic differentiation in vitro. And also, Osteogenic differentiation of hBMSCs was carried out using osteogenic medium containing 50 mg/ml ascorbic acid (Sigma-Aldrich, St. Louis, MO, USA), 5 mM β-glycerophosphate (Sigma-Aldrich) and 10 nM dexamethasone (Sigma-Aldrich).

### Cell transfection

Short hairpin RNA targeting LOC100506178 (shLOC100506178), miR-214-5p mimics, miR-214-5p inhibitor and negative control were synthesized by GenePharma (Shanghai, China). The full-length LOC100506178 was sequentially amplified from complementary DNA (cDNA) and cloned into the downstream of pcDNA4.0 vector to generate LOC100506178 overexpression plasmid, and the sequence was confirmed by DNA sequencing (Sangon, Shanghai, China). 1 × 10^4^ cells per well were seeded in a 96-well plate and incubated for 24 h, then cells were transfected with RNAs (100 nM) or plasmids (20 ng) using Lipofectamine 2000 (Invitrogen, San Diego, USA) in serum-free medium in accordance with the manufacturer’s instructions. The cells with more than 80% transfection efficiency were used for further study and followed by osteogenic differentiation.

### Alizarin Red S Staining

Alizarin Red S Staining was performing on day 28 of osteoblast differentiation. Briefly, hBMSCs were washed with PBS and fixed with 70% ice-cold ethanol for 30 min at room temperature. Then, cells were stained with 2% Alizarin red S staining kit (Sigma, St. Louis, Missouri) for 30 min at room temperature. Subsequently, cells were washed with water and observed under an inverted microscope. About 10% acetic acid was used to extract the alizarin red S in cell culture, then 10% ammonium hydroxide was used to neutralize the acid. The absorbance of cell culture extractions and alizarin red S standard at 550 nm were read with a plate reader.

### Alkaline phosphatase (ALP) activity

ALP activity was analyzed using the BioVision ALP activity colorimetric assay kit (BioVision Inc., Milpitas, CA) according to the manufactures’ instructions. In brief, hBMSCs were cultured for 3, 7, 14 and 28 days and fixed with 4% formaldehyde in 90% ethanol for 30 s at room temperature. Then, cells were incubated with p-nitrophenyl phosphate solutionfor 45 min. The optical densities were measured using a spectrophotometer plate reader.

### Quantitative real-time PCR (RT-PCR) assay

The total RNAs from hBMSCs were extracted by using TRIzol reagent (Takara, Japan). Reverse transcription was performed by using BestarTM qPCR RT kit (DBI Bioscience, China) with 5 µg of total RNAs. qRT-PCR amplification reaction was performed by using the Reverse Transcription kit (Takara, Japan) on an ABI7500 system. The primers were obtained from BGI (Shenzhen, China). The relative gene expression was calculated by 2^−ΔΔCq^ method. Relative lncRNA expression and mRNA were normalization to GAPDH, while relative miRNA was normalized to U6.

### Western Blot assay

The treated hBMSCs were lysed with RIPA buffer according to the manufacturer’s instructions, and the concentration of proteins was determined by using a BCA kit (Pierce, Rockford). Total proteins were separated by 10% SDS-PAGE and transferred into polyvinyldifluoridine (PVDF) membranes (Millipore, U.S.A.).The membranes were blocked with 5% nonfat milk for 1 h at room temperature, and then were subjected to the incubation overnight with primary antibody that against BMP2 (ab14933, abcam, Cambridge, UK) and GAPDH (1:10000, sc420485, Satna Cruz). Next day, after washing, the membranes were incubated with secondary peroxidase-conjugated anti-mouse or anti-rabbit antibodies (Zhong Shan Golden Bridge Biotech Company, China) for 1.5 h at room temperature. Finally, the results were measured by the enhanced chemiluminescence substrate kit (Amersham Biosciences Inc., Piscataway, New Jersey, USA) on an ECL system (Amersham Pharmacia, Piscataway, NJ, USA).

### Luciferase reporter assays

The targets of LOC100506178 were predicted by LncBase Predicted v2 and found miR-214-5p contains binding site for LOC100506178. TargetScan and miRDB predicted BMP2 is a target gene of miR-214-5p. Plasmids contain wild-type or mutant LOC100506178 and miR-214-5p, and wild-type or mutant BMP2 and miR-214-5p, were constructed with pmirGLO luciferase vectors (Promega, Madison, Wisconsin). For luciferase reporter assay, approximately 1 × 10^4^ 293T cells were plated in 24-well plate and co-transfected with miRNAs and these above luciferase plasmids using Lipofectamine 2000 (Invitrogen). After 48 h transfection, relative luciferase activity was determined using Dual Luciferase Reporter Assay System (Promega) with Renilla luciferase activity as an internal control.

### RNA immunoprecipitation (RIP)

RIP experiments were conducted using the Magna RIP RNA-Binding Protein Immunoprecipitation Kit (Millipore, Billerica, Massachusetts) with an anti-AGO1 antibody (Millipore). In brief, cell extract was prepared by RIP lysis buffer and incubated with magnetic beads conjugated with human anti-AGO1 antibody (Millipore) or normal IgG as negative control. Subsequently, quantitative real-time PCR was performed to detect the relative expression of LOC100506178 and miR-214-5p as described above.

### In vivo ectopic bone formation assay

All procedures were approved by the Institutional Animal Care and Use Committee of Sichuan Provincial People’s Hospital. Six-week-old female BALB/C-nu mice were obtained from Beijing Vital River Laboratory Animal Technology Co., Ltd (Beijing, China) and housed in SPF conditions. The hBMSCs were transfected with LOC100506178 alone or together with miR-214-5p mimics or NC. 5 × 10^6^ transfected cells were mixed with 10 mg of deproteinized bovine bone (Bio-Oss, Geistlich, Wolhusen, Switzerland) and subcutaneously transplanted on the back of mice. After eight weeks, the transplants were collected and fixed with 4% paraformaldehyde, embedded in paraffin and sectioned into 4 µm. Then the sections were de-paraffinized and stained with hematoxylin and eosinfor histology analysis.

### Statistical analysis

The statistical data were expressed as mean ± standard deviations (SD) form at least in triplicate and statistical analysis was performed using SPSS 21.0 statistical software. The unpaired Student’s *t*-test or one-way analysis of variance (ANOVA) was used to assess differences among groups and *p* value less than 0.05 was considered as the statistical significance.

## Results

### LOC100506178 is increased in BMP2-induced osteogenic differentiation of hBMSCs

**Figure 1:**
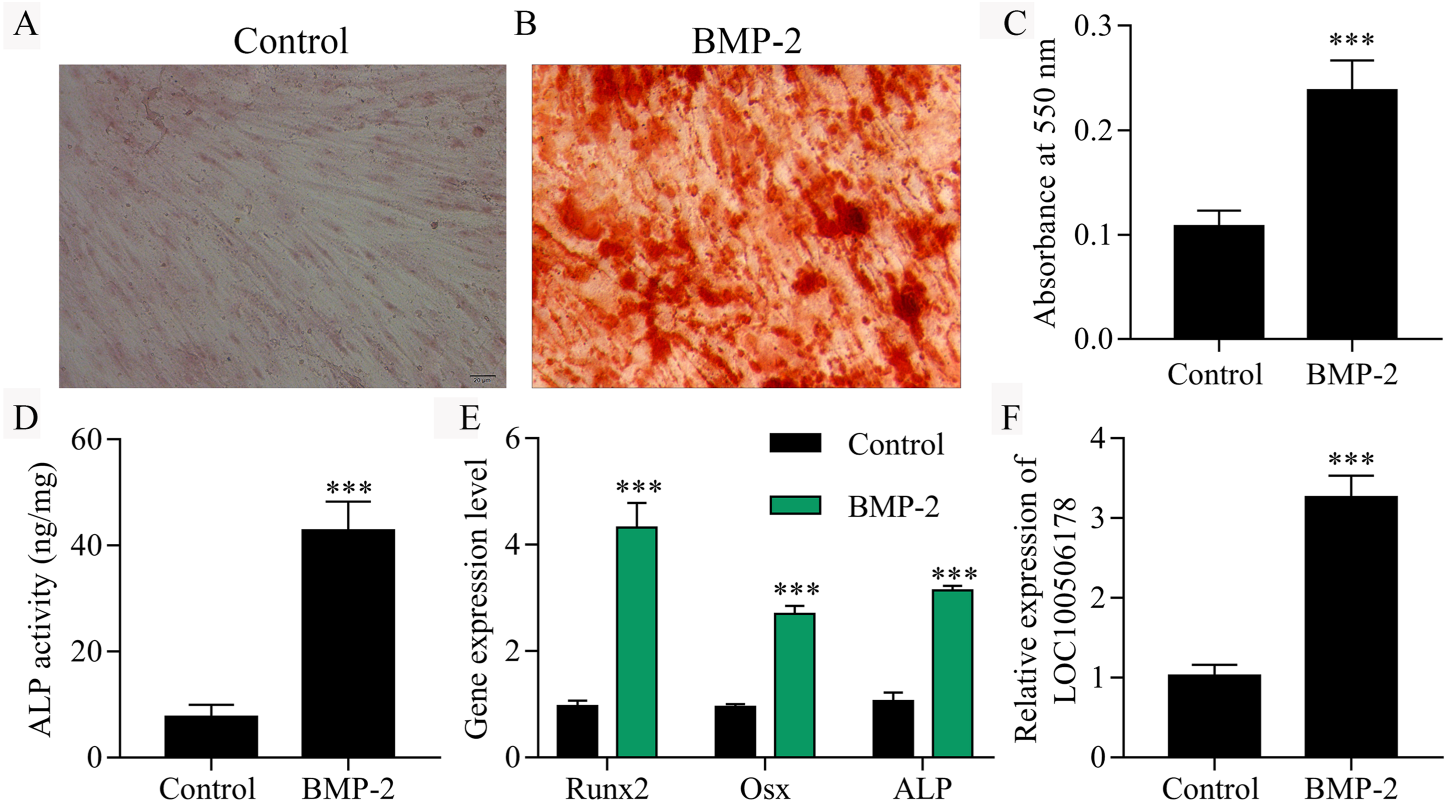
LOC100506178 is increased in BMP2-induced osteogenic differentiation of hBMSCs. (A, B) Alizarin Red S Staining was performing on day 28 of osteoblast differentiation. (C) To quantify the amount of alizarin red staining in different groups, ^∗∗∗^*p* < 0.001. (D) Quantitative evaluation of the osteogenic differentiation capacity using alkaline phosphatase (ALP) activity assay, ^∗∗∗^*p* < 0.001. (E) The expression levels of RUNX2, Osx and ALP mRNA were examined by qPCR, ^∗∗∗^*p* < 0.001. (F) qPCR results also showed that the expression of LOC100506178 was significantly up-regulated in BMP2 stimulated hBMSCs, ^∗∗∗^*p* < 0.001.

The osteogenic differentiation process was triggered in hBMSCs by incubation with BMP2. Firstly, we observed an enhancement of mineralized bone matrix in hBMSCs after BMP2 treatment assessed by Alizarin Red S Staining (Figs. 1A–1C). As a typical osteoblastic phenotype, the ALP activity was significantly increased in BMP2-induced hBMSCs compared with non-induced groups (Fig. 1D). Moreover, we analyzed the expression of reliable markers for osteoblastic differentiation, inlcuding RUNX2, Osx and ALP by qPCR. Our results showed that BMP2 significantly increased the expression of RUNX2, Osx and ALP in BMP2-induced hBMSCs compared with un-treated cells (Fig. 1E). Furthermore, qPCR results also showed that the expression of LOC100506178 was significantly up-regulated in BMP2 stimulated hBMSCs (Fig. 1F) and the expression of LOC100506178 was increased in ascorbic acid and beta-glycerophosphate induced osteogenic differentiation of hBMSCs (Fig. S1), which indicated that LOC100506178 might play an important role in BMP2-induced osteogenic differentiation.

### LOC100506178 promotes BMP2-induced osteogenic differentiation of hBMSCs

**Figure 2:**
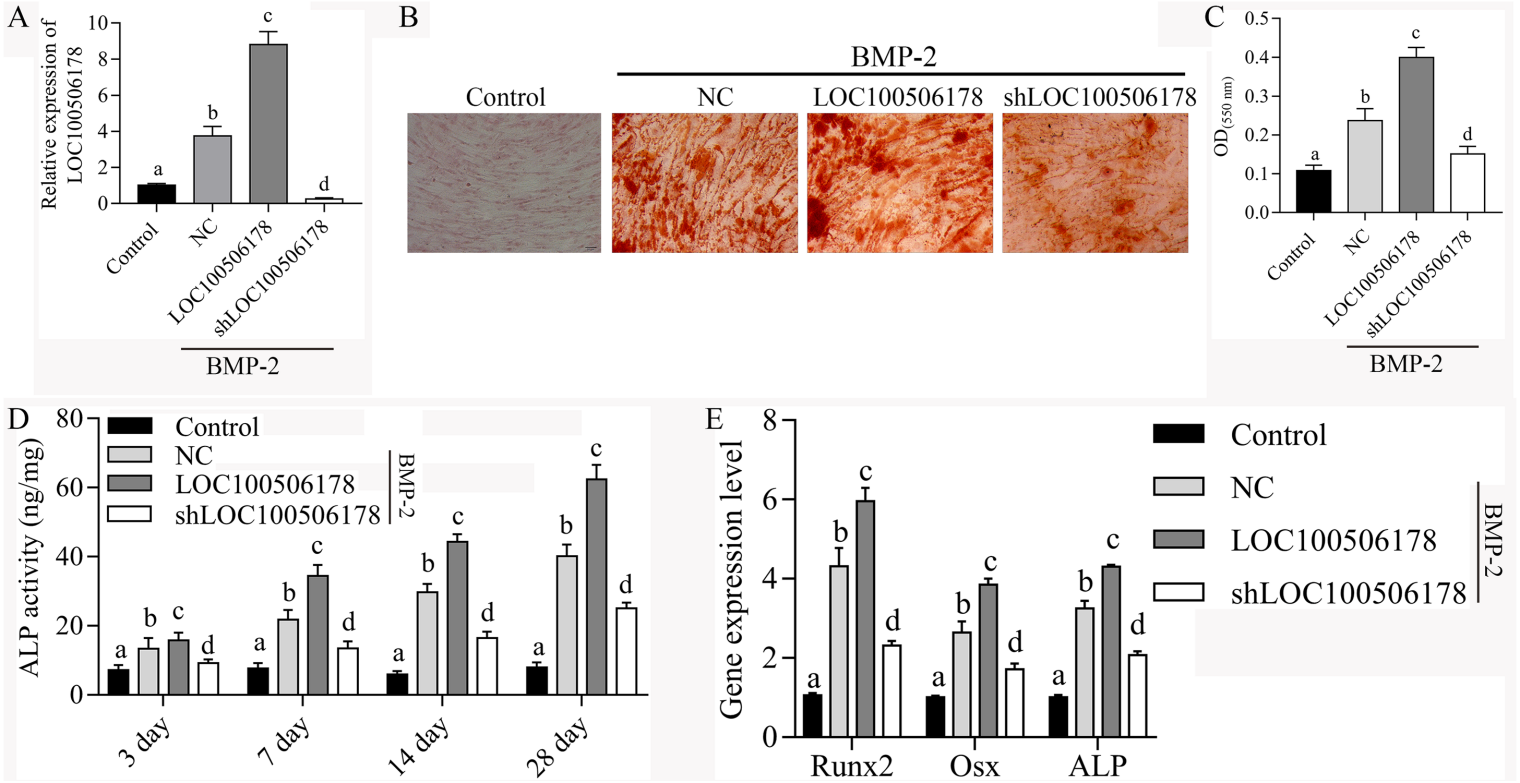
LOC100506178 promotes BMP2-induced osteogenic differentiation of hBMSCs. (A) qPCR analyzed the expression of LOC100506178 in hBMSCs after transfection of LOC100506178 overexpression plasmid and LOC100506178 knockdown plasmid, different letters mean significantly difference in different groups. (B) Alizarin Red S Staining was performing in hBMSCs on day 28 after induction. (C) To quantify the amount of alizarin red staining in different groups, different letters mean significantly difference in different groups. (D) Quantitative evaluation of the osteogenic differentiation capacity using alkaline phosphatase (ALP) activity assay during osteogenesis differentiation, different letters mean significantly difference in different groups. (E) The mRNA expression of RUNX2, Osx and ALP was measured in BMP2-induced hBMSCs transfected with LOC100506178. Different letters mean significantly difference in different groups.

To confirm whether LOC100506178 contributes to BMP2-induced osteogenic differentiation of hBMSCs, LOC100506178 overexpression plasmids and shLOC100506178 plasmids were transfected into the hBMSCs to evaluate the expression of LOC100506178 on the BMP-2-induced osoteoblstic differentiation. As demonstrated by qPCR, the expression of LINC00968 was significantly increased in hBMSCs transfected with LOC100506178 overexpression plasmids, while decreased in hBMSCs transfected with shLOC100506178 plasmids (Fig. 2A). Alizarin Red S Staining results showed that the mineralized bone matrix was obviously enhanced after LINC00968 overexpression in BMP2-induced hBMSCs, while weakened in BMP2-induced hBMSCs after LINC00968 knockdown (Fig. 2B and 2C). Overexpression of LOC100506178 also led to increased ALP activity and knockdown of LOC100506178 suppressed ALP activity during BMP2-induced hBMSCs osteogenesis differentiation (Fig. 2D). What’s more, Overexpression of LOC100506178 promoted the expression of RUNX2, Osx and ALP and knockdown of LOC100506178 inhibited the expression of RUNX2, Osx and ALP in BMP2-induced hBMSCs (Fig. 2E). Our data indicated that LOC100506178 contributes to BMP2-induced osteogenic differentiation of hBMSCs.

### LOC100506178 functions as an endogenous sponge of miR-214-5p

**Figure 3:**
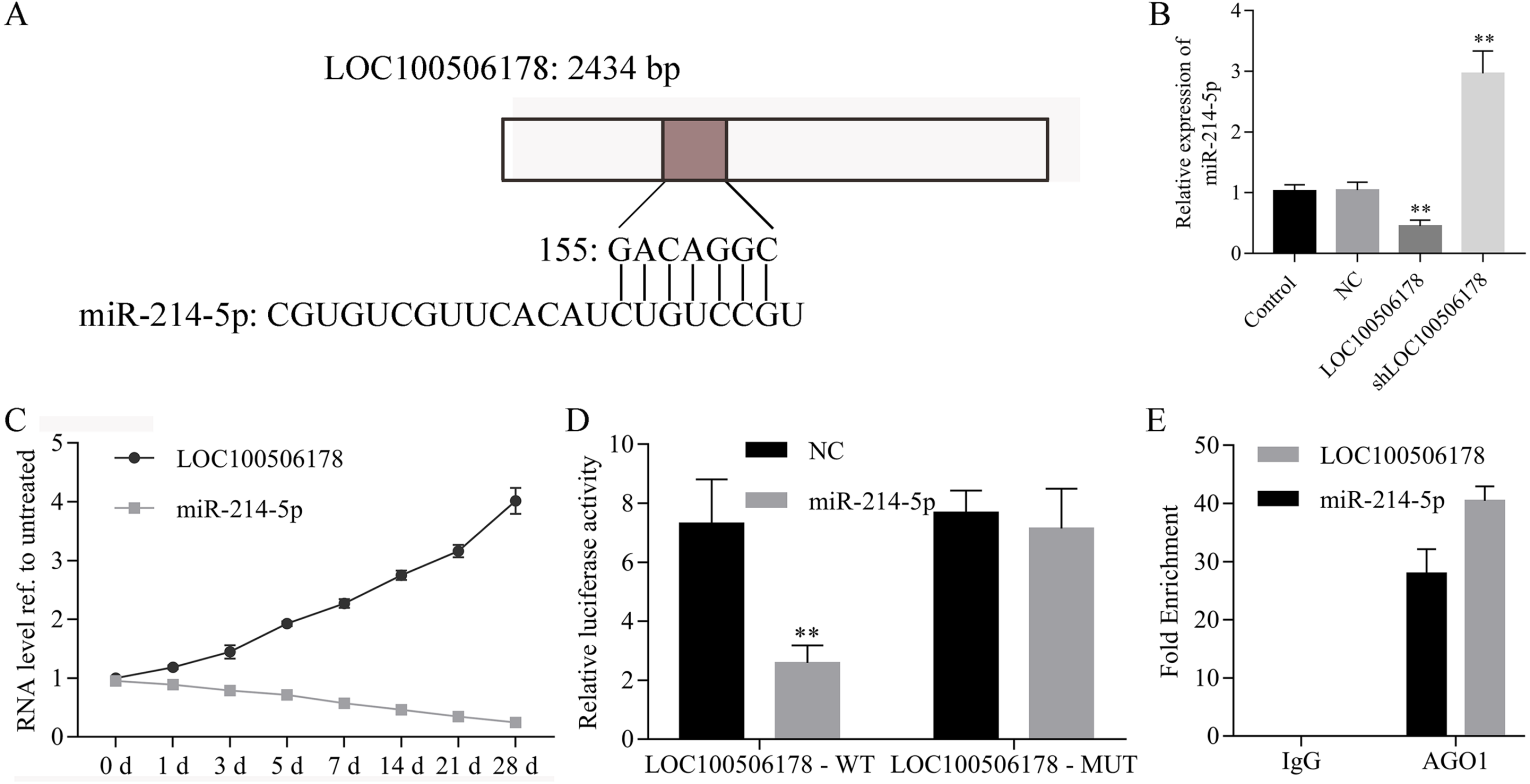
LOC100506178 functions as an endogenous sponge of miR-214-5p. (A) Putative binding sites of miR-214-5p in LOC100506178 were shown. (B) miR-214-5p was increased in shLOC100506178 transfected hBMSCs and decreased in LOC100506178 overexpression plasmids transfected hBMSCs, ^∗∗^*p* < 0.01. (C) Correlation analysis between LOC100506178 and miR-214-5p levels in hBMSCs at 0, 1, 3, 5, 7, 14 and 28 d after osteogenic differentiation. (D) Luciferase reporter assay showed the directly reaction between LOC100506178 and miR-214-5p. (E) RIP assay demonstrated that LOC100506178 and miR-214-5p expression levels were significantly higher in the anti-AGO1 group compared with the anti-normal IgG group.

To explore the underlying molecular mechanism by which LOC100506178 regulated osteogenic differentiation, predicted targets of LOC100506178 were analyzed using LncBase Predicted v2 software. As expected, miR-214-5p might be the potential target of LOC100506178 with higher predictive score (Fig. 3A). Then, we analyzed the expression of miR-214-5p in LOC100506178 or shLOC100506178 transfected hBMSCs. As shown in Fig. 3B, miR-214-5p expression was significantly decreased in LOC100506178 transfected hBMSCs, while was significantly increased in shLOC100506178 transfected hBMSCs. Furthermore, we analyzed the association between LOC100506178 and miR-214-5p during the process of osteogenic differentiation from day 0 to day 28. Our results showed that the expression of miR-214-5p negatively correlated with the expression of LOC100506178 (Fig. 3C). The directly reaction between LOC100506178 and miR-214-5p was measured by luciferase reporter assay. As showed in Fig. 3D, the luciferase activity of LOC100506178 wild-type reporter was strongly suppressed by miR-214-5p overexpression. However, the LOC100506178 mutant reporter was not affected by miR-214-5p mimics. RIP assay further demonstrated that LOC100506178 and miR-214-5p expression levels were significantly higher in the anti-AGO1 group compared with the anti-normal IgG group (Fig. 3E). These results indicated that LOC100506178 directly regulates the expression of miR-214-5p.

### miR-214-5p inhibits BMP2-induced osteogenic differentiation of hBMSCs

**Figure 4:**
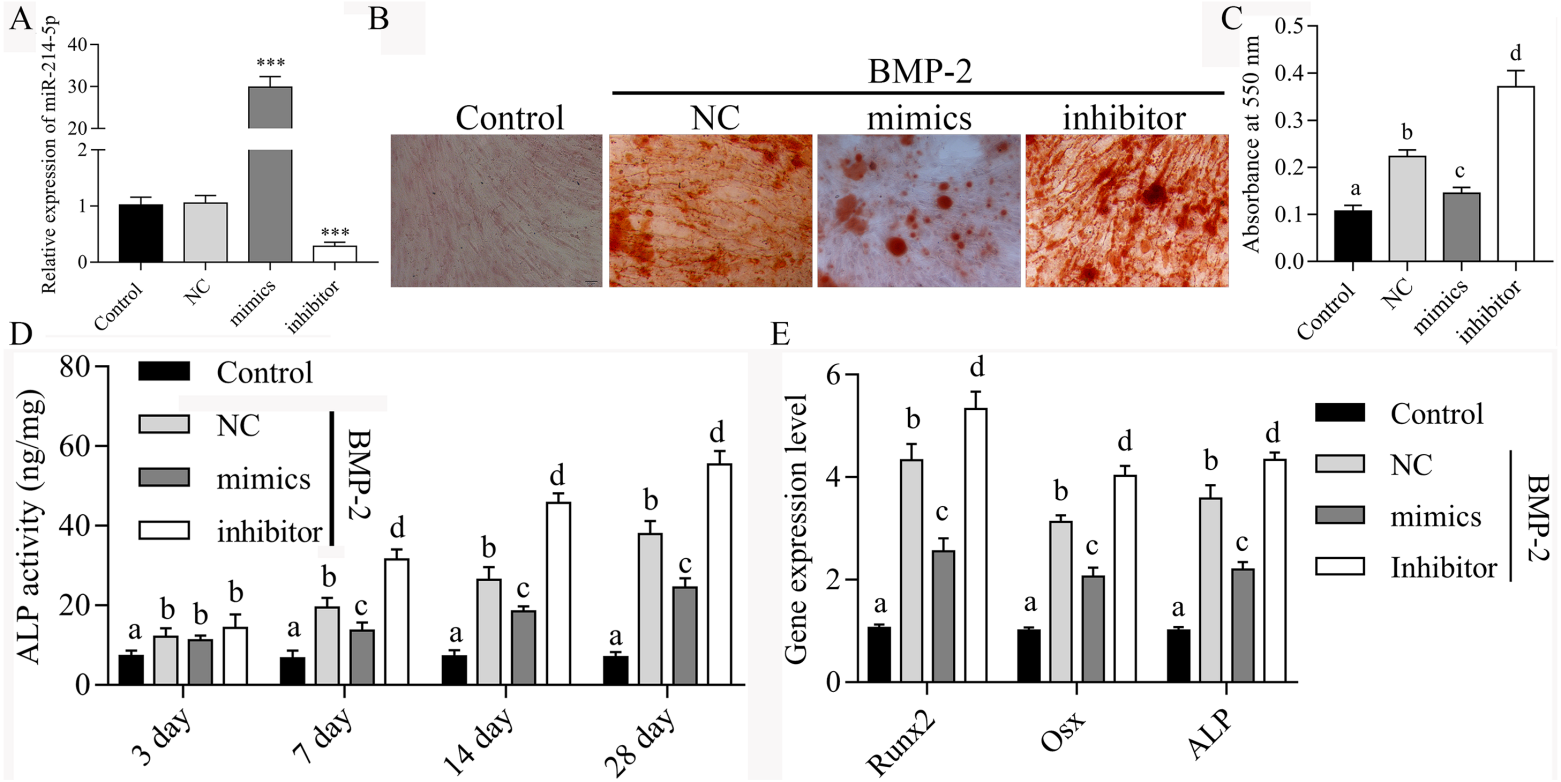
miR-214-5p inhibits BMP2-induced osteogenic differentiation of hBMSCs. (A) qPCR analyzed the expression of miR-214-5p in hBMSCs, ^∗∗∗^*p* < 0.001. (B) Alizarin Red S Staining was performing in hBMSCs on day 28 after induction. (C) To quantify the amount of alizarin red staining in different groups, different letters mean significantly difference in different groups. (D) Quantitative evaluation of the osteogenic differentiation capacity using alkaline phosphatase (ALP) activity assay during osteogenesis differentiation, different letters mean significantly difference in different groups. (E) Overexpression of miR-214-5p suppressed the expression of RUNX2, Osx and ALP and knockdown of miR-214-5p promoted the expression of RUNX2, Osx and ALP in BMP2-induced hBMSCs, different letters mean significantly difference in different groups.

To confirm the effects of miR-214-5p on BMP2-induced osteogenic differentiation of hBMSCs, the expression of miR-214-5p was manipulated in hBMSCs during BMP-2-induced osoteoblstic differentiation. As demonstrated by qPCR, the expression of miR-214-5p was significantly increased in hBMSCs transfected with miR-214-5p mimics, while decreased in hBMSCs transfected with miR-214-5p inhibitor (Fig. 4A). Alizarin Red S Staining results showed that the mineralized bone matrix was obviously reduced after miR-214-5p overexpression in BMP2-induced hBMSCs, while enhanced in BMP2-induced hBMSCs after miR-214-5p inhibitor transfection (Figs. 4B and 4C). Overexpression of miR-214-5p led to inhibit ALP activity and knockdown of miR-214-5p increased ALP activity in BMP2-induced hBMSCs during osteoblast differentiation (Fig. 4D). What’s more, Overexpression of miR-214-5p suppressed the expression of RUNX2, Osx and ALP and knockdown of miR-214-5p promoted the expression of RUNX2, Osx and ALP in BMP2-induced hBMSCs (Fig. 4E). Our data indicated that miR-214-5p contributes to BMP2-induced osteogenic differentiation of hBMSCs.

### LOC100506178 positively regulates BMP2 expression through sponging miR-214-5p

**Figure 5:**
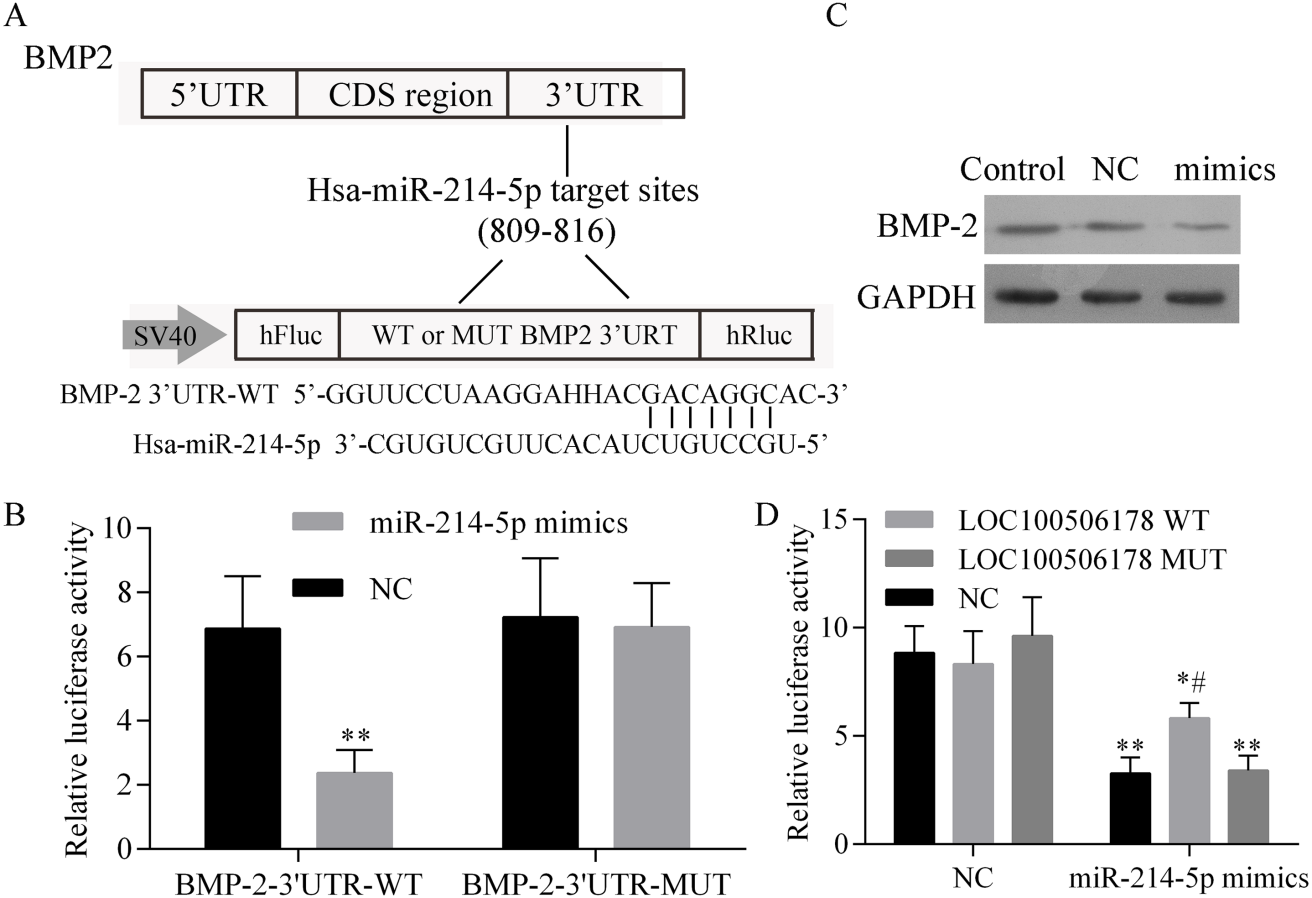
LOC100506178 positively regulates BMP2 expression through sponging miR-214-5p. (A) Schematic diagram of miR-214-5p target site in the 3′UTR of BMP2. (B) The BMP2-WT 3′UTR luciferase reporter activity was significantly decreased by miR-214-5p mimics, which was abolished in the mutation of BMP2 3′UTR, ^∗∗^*p* < 0.01 compared with BMP-2-3′UTR-WT+NC. (C) Western blot showed that endogenous BMP2 protein level was obviously suppressed by miR-214-5p mimics transfection. (D) The luciferase activity of BMP2, which was inhibited by miR-214-5p mimics, was significantly rescued after co-transfection of LOC100506178 WT, compared with vector or LOC100506178 MUT. ^∗^*p* < 0.05, ^∗∗^*p* < 0.01, compared with NC + vector, NC + LOC100506178 WT or NC + LOC100506178 MUT; #*p* < 0.05, compared with miR-214-5p mimics + LOC100506178 MUT or miR-214-5p mimics + vector.

Firstly, BMP2 was predicted to be a target gene of miR-214-5p and the predicted binding sites of miR-214-5p in BMP2 were shown in Fig. 5A. A luciferase assay was then employed to identify the miR-214-5p target region of BMP2 3′UTR in 293T cells. As shown in Fig. 5B, the BMP2-WT 3′UTR luciferase reporter activity was significantly decreased by miR-214-5p mimics, which was abolished in the mutation of BMP2 3′UTR. We also analyzed the protein expression levels of endogenous BMP2 in response to miR-214-5p and found endogenous BMP2 protein levels were obviously suppressed by miR-214-5p mimics transfection (Fig. 5C), which indicated that BMP2 is a target gene of miR-214-5p. Besides, 293T cells were transfected with miR-214-5p mimics or NC together with the BMP2 luciferase reporter and LOC100506178 WT, LOC100506178 MUT or vector. As shown in Fig. 5D, the luciferase activity of BMP2 was significantly suppressed by miR-214-5p mimics, no matter 293T cells transfected with BMP2 luciferase reporter and, LOC100506178 MUT or Vector. Nevertheless, in comparison with LOC100506178 MUT and the vector, transfection of LOC100506178 WT could significantly restore the activity of BMP2 luciferase. Taken together, these results suggested that LOC100506178 could protect BMP2 from miR-214-5p-mediated degradation.

### LOC100506178 increases in vivo ectopic bone formation in hBMSCs by suppression miR-214-5p

**Figure 6:**
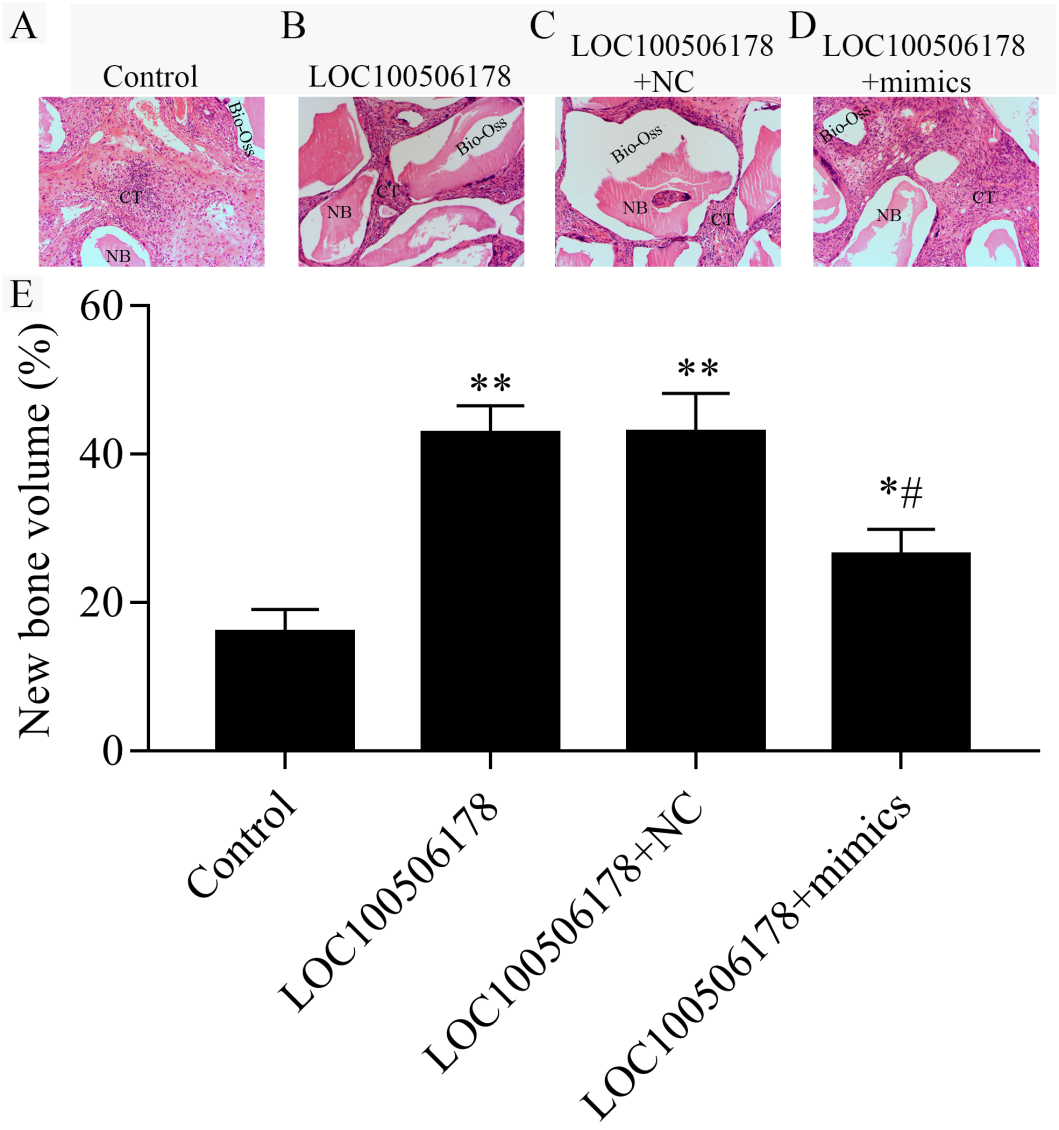
LOC100506178 increases in vivo ectopic bone formation in hBMSCs by suppression miR-214-5p. (A–D) Representative image of transplanted specimens in different treated groups by H&E staining; NB, new bone; CT, connective tissue. (B) New bone volume was quantified as per total specimen area. The data represent the mean ± SD, ^∗^*p* < 0.05, ^∗∗^*p* < 0.01, compared with control; ^#^*p* < 0.05, compared with LOC100506178.

The results described above indicated that LOC100506178 may function as miR-214-5p sponges to positively regulate the osteogenic differentiation of hBMSCs in vitro, we next explored whether the regulation of LOC100506178 and miR-214-5p in hBMSCs also exerts an effect on in vivo bone formation. To induce ectopic bone formation, hBMSCs were transfected with LOC100506178 overexpression plasmid alone or together with miR-214-5p mimics or NC, followed by subcutaneously transplanted in nude mice. After eight-week transplantation, we observed regenerated new bone and nodes in transplants. Compared with control group, LOC100506178-transfected hBMSCs showed significantly induced ectopic bone formation. However, co-transfection of LOC100506178 overexpression plasmid and miR-215-5p mimics impaired bone formation in compared with the LOC100506178 overexpression plasmid transfection alone group (Fig. 6). These results demonstrated that LOC100506178 could promote ectopic bone formation, which was reversed by miR-214-5p.

## Discussion

BMSC differentiation is precisely orchestrated and regulated by molecular and mechanical signals that have attracted great attentions in recent years (Chen & Shou, 2016; Fan et al., 2016; Liu et al., 2016; Yi et al., 2017). Therefore, understanding the molecular mechanisms underlying BMSC differentiation and modulating lineage commitment of BMSCs is of importance for therapeutic purposes in different diseases (Forbes & Rosenthal, 2014).

Intensive studies have demonstrated that a number of critical signaling pathways, including TGF- β/BMP signaling, Wnt signaling, Shh signaling et al., are involved in regulating the lineage commitment of BMSCs (Jiang et al., 2013; Liu et al., 2011; Zhen et al., 2013). LncRNAs are found to be involved in a large range of biological processes, which are correlated with human life processes and various diseases. Emerging evidence is revealing that lncRNAs contribute to the differentiation process of stem cells (Hu & Shan, 2016). Recent studies found that some lncRNAs have been found to play as key regulators in osteogenic differentiation of BMSCs (Qu et al., 2016; Song et al., 2015). However, the role of lncRNA LOC100506178 on the osteogenic differentiation process of BMSCs known little. LncRNA HOTAIRM1 was identified as a critical regulator to promote osteogenesis of MSCs though positively modulates the activity of JNK and c-Jun signaling pathway (Liu et al., 2019). Zheng et al. (2019) found that lncRNA MALAT1 suppressed osteogenic differentiation of BMSCs via regulating the MAPK signaling pathway-related proteins extracellular signal-regulated kinase 1/2 (ERK1/2) and P38. Here, in our present study, we found that lncRNA LOC100506178 plays as a key regulator in BMP2-induced osteogenic differentiation of hBMSCs.

There is now evidence that lncRNAs regulate gene expression and function by competing with miRNAs for binding to target mRNAs. Here, we found LOC100506178 functions as an endogenous sponge of miR-214-5p to up-regulated BMP2 expression. Previously studies have shown that the expression of miR-214 is significantly decreased during osteogenic induction. First, overexpression of miR-214 inhibits BMP2 expression by binding to its 3′UTR; whereas, overexpression of lncRNA KCNQ1OT1 increased BMP2 expression by suppressing miR-214 expression to promote osteogenic differentiation of BMSCs (Wang et al., 2019). Subsequently, overexpression of miR-214 inhibits osteoblast differentiation of BMSCs by suppressing ALP activity and gene expression of OCN, type I collagen (Col I), and osteopontin (OPN) and also inhibiting JNK and p38 pathways (Guo et al., 2017). A later study showed that overexpression of miR-214-5p inhibited Runx2, ALP, and collagen alpha-1 (I) chain (COL1A1) expression and attenuated the osteogenic differentiation of BMSCs (Qiu et al., 2018). In our present study, we found that overexpression of miR-214-5p suppressed the expression of RUNX2, ALP and Osx, and inhibited osteogenic differentiation of hBMSCs through inbhiting BMP2 expression by binding to its 3′UTR. Our data suggested that LOC100506178 promoted osteogenic differentiation and bone formation through competitively binding to miR-214-5p to up-regulate BMP2 expression.

## Conclusions

In our present study, we firstly demonstrated that LOC100506178 is increased during osteogenic differentiation of hBMSCs, which acts as a positive regulator of osteoblast differentiation via positively regulating BMP2 expression through sponging miR-214-5p. We also showed that the function promotion of LOC100506178 can accelerate in vivo bone formation by down-regulating miR-214-5p expression. Our data indicated a novel molecular pathway LOC100506178/miR-214-5p/BMP2 in relation to hBMSCs differentiation into osteoblasts, which may facilitate bone anabolism.

##  Supplemental Information

10.7717/peerj.8909/supp-1 Figure S1 LOC100506178 is increased in ascorbic acid and beta-glycerophosphate induced osteogenic differentiation of hBMSCs (A) Alizarin Red S Staining was performing on day 28 of osteoblast differentiation. (B) qPCR results also showed that the expression of LOC100506178 was significantly up-regulated in ascorbic acid and beta-glycerophosphate stimulated hBMSCs. ^∗∗∗^*p* < 0.001.

10.7717/peerj.8909/supp-2 Supplemental Information 2 Raw Data

## Additional Information and Declarations

### Data Availability

The following information was supplied regarding data availability: The raw measurements are available in the Supplemental Files.
