# Supplementary figures and images for: LncRNA LOC100506178 promotes osteogenic differentiation via regulating miR-214-5p-BMP2 axis in human bone marrow mesenchymal stem cells
Source: PeerJ. 2020 Apr 15;8:e8909. doi: 10.7717/peerj.8909 (PMC7166045; doi:10.7717/peerj.8909)

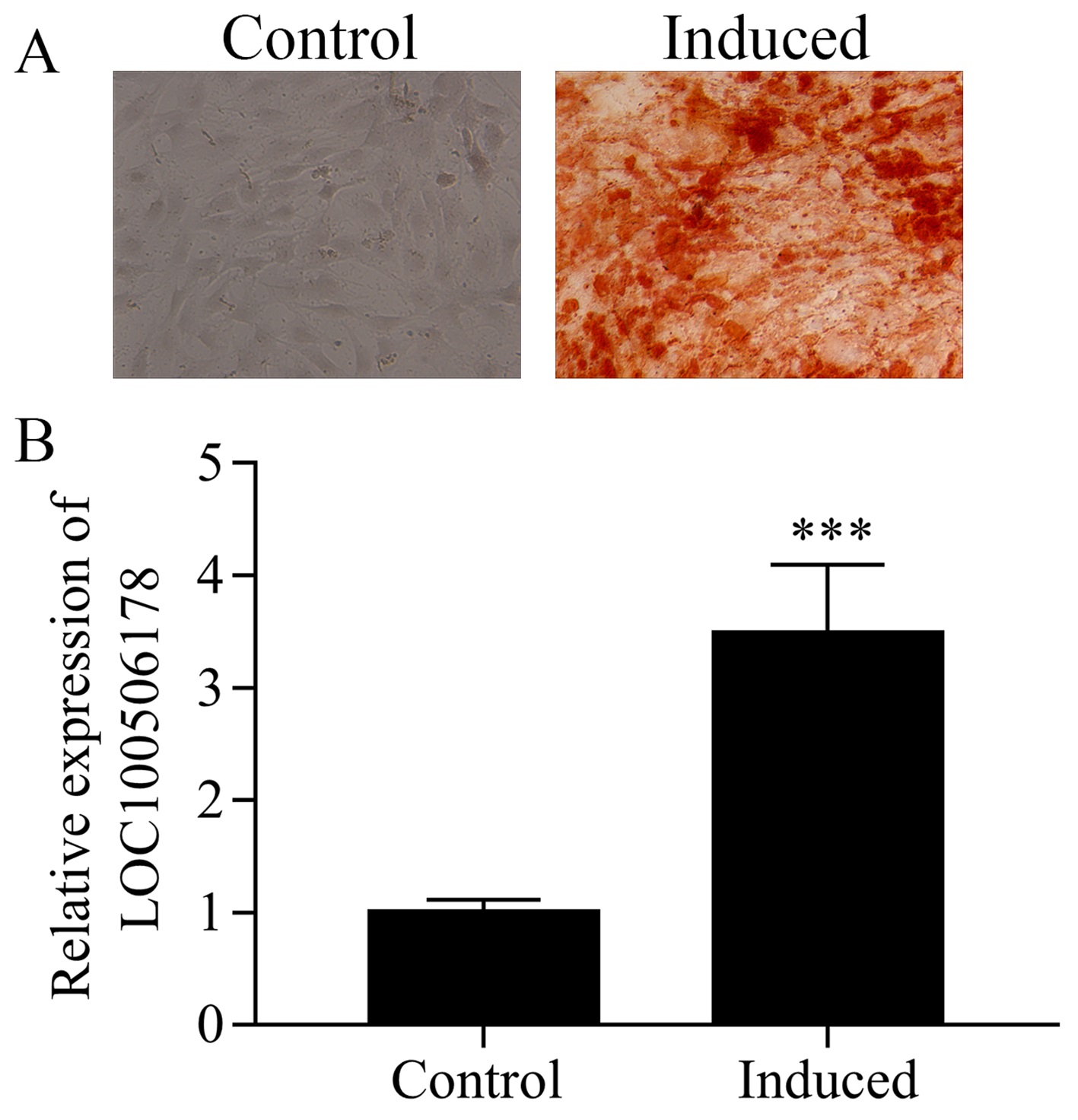

Supplement: Figure S1 — (A) Alizarin Red S Staining was performing on day 28 of osteoblast differentiation. (B) qPCR results also showed that the expression of LOC100506178 was significantly up-regulated in ascorbic acid and beta-glycerophosphate stimulated hBMSCs. ∗∗∗p < 0.001. [file peerj-08-8909-s001.jpg]
